# Significance of baseline and change in neutrophil-to-lymphocyte ratio in predicting prognosis: a retrospective analysis in advanced pancreatic ductal adenocarcinoma

**DOI:** 10.1038/s41598-017-00859-5

**Published:** 2017-04-09

**Authors:** Yang Chen, Huan Yan, YanRong Wang, Yan Shi, GuangHai Dai

**Affiliations:** Medical Oncology department 2, Chinese PLA General Hospital and Chinese PLA Medical School, Beijing, 100853 China

## Abstract

The neutrophil-lymphocyte ratio (NLR) has been reported to be associated with prognosis in several cancers. The objective of our study was to evaluate the prognostic role of baseline NLR and change in NLR (ΔNLR) in advanced pancreatic cancer underwent chemotherapy. Between January 2010 and June 2015, 132 patients underwent chemotherapy were eligible for assessment. Based on our patients’ data, the cut-off value of NLR was 2.78 according to receiver operating characteristic curve. We observed that a high level of baseline NLR (NLR > 2.78) was a poor prognostic factor for overall survival (multivariable hazard ratio [HR] = 2.648, P < 0.001). Increased NLR (ΔNLR > 0) after 2 cycles of chemotherapy was associated with higher risk compared to ΔNLR ≤ 0 (multivariable HR = 1.894, P = 0.007). Combining both NLR and ΔNLR factors, multivariate analysis showed a significant higher risk (HR = 5.817, P < 0.001) for patients with high baseline NLR and increased NLR after 2 cycles of chemotherapy compared to patients with low baseline NLR and ΔNLR ≤ 0. In conclusion, both baseline NLR and ΔNLR are independent prognostic predictors for patients with advanced pancreatic cancer underwent chemotherapy.

## Introduction

Pancreatic cancer is the fourth leading cause of cancer death in United States for both men and women^1^. Cases are usually diagnosed at an advanced stage with limited treatment options available. The 5-year survival rate is as low as <5%. The Karnofsky performance status (KPS) is usually worse in advanced stage pancreatic cancer, and precise patient selection for effective treatment is still unmet clinical need. There is lack of means to predict prognosis. Although pathological stage is known as a good predictor, it is difficult to obtain tumour tissue in most of the patients. Recently, blood neutrophil-to-lymphocyte ratio (NLR) was shown valuable to predict prognosis in cancer patients, as inflammatory responses play decisive roles at different stages of tumour development, including initiation, promotion, invasion, and metastasis^2, 3^. The tumour microenvironment contains innate immune cells (including macrophages, neutrophils, mast cells, myeloid-derived suppressor cells, dendritic cells, and natural killer cells) and adaptive immune cells (T and B lymphocytes) in addition to the cancer cells and their surrounding stroma. The net outcome of therapy-induced inflammation is controversial, as on the one hand it can have tumour-promoting functions just like the necrosis that accompanies rapid tumour growth^4^, but on the other hand it can enhance the cross-presentation of tumour antigens and subsequent induction of an anti-tumour immune response^5^.

Biomarkers of inflammation such as blood NLR and blood platelet-to-lymphocyte ratio (PLR) have been associated with clinical outcomes in a number of tumours including colorectal cancer, gastric cancer, non-small cell lung cancer, small cell lung cancer and pancreatic cancer^6–9^. However, neutrophil and lymphocyte count may be influenced by a host of clinical factors such as KPS, age, previous treatments, coexisting infection, and impaired renal or hepatic function. Chemotherapy could have a significant impact on patients’ inflammation environment. There were limited studies focused on the change in NLR (ΔNLR) when patients underwent chemotherapy. In this study, we investigated the prognostic role of both baseline NLR and ΔNLR in patients with advanced pancreatic adenocarcinoma underwent chemotherapy.

## Results

**Figure 1:**
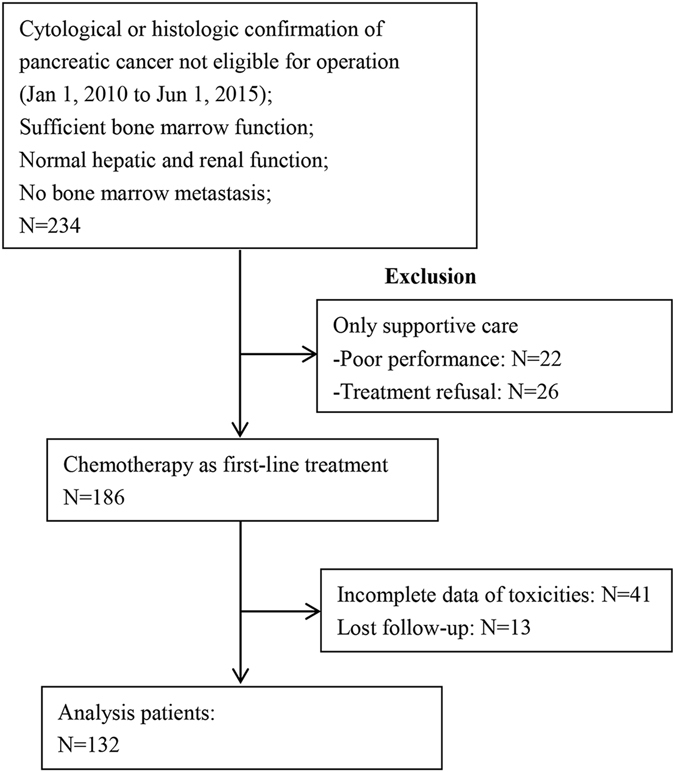
Study flow chart.

Total 132 patients (85 male and 47 female) with histologically confirmed advanced pancreatic cancer who received chemotherapy were eligible for assessment (Fig. 1). The median age at diagnosis was 57 (95% CI: 41.6–69.7). By July 30, 2016, 116 (87.9%) patients passed away, and the median survival was 7.66 months (95% CI: 6.13–9.23). Of the 31 (23.5%) patients were locally advanced and 101 (76.5%) patients had distant metastasis. We used 1-year survival as the time point to generate the receiver operating characteristic (ROC) curve and determined the optimal cut-off value of 2.78 for baseline NLR with the area under the curve (AUC) of 0.634. We used baseline NLR and NLR after 2 cycles to determine ΔNLR and categorized patients into ΔNLR ≤ 0 group and ΔNLR > 0 group (Table 1).

**Table 1:** Clinical characteristics of advanced pancreatic cancer patients.

Characteristics	N (%) (n = 132)
**Gender**	
Male	85 (64.4)
Female	47 (35.6)
**Age**	
>60	41 (31.1)
≤60	91 (68.9)
**KPS**	
90	108 (81.8)
80	20 (15.2)
70	4 (3)
**Location**	
Head	46 (34.8)
Body/tail	86 (65.2)
**Stage at diagnosis**	
Locally advanced	31 (23.5)
Distant metastasis	101 (76.5)
**Albumin (g/L)**	
≤35	11 (8.3)
>35	121 (91.7)
**Baseline WBC (×10** ^**9**^ **/L)**	
median (range)	6.515 (4.39–11.88)
**Baseline Hemoglobin (g/L)**	
median (range)	129 (101.65–156.35)
**Baseline Platelets (×10** ^**9**^ **/L)**	
median (range)	191.5 (106–311.5)
**WBC after 2 cycles (×10** ^**9**^ **/L)**	
median (range)	5.625 (2.78–13.68)
**Hemoglobin after 2 cycles (g/l)**	
median (range)	111 (87.65–135.4)
**Platelets after 2 cycles (×10** ^**9**^ **/L)**	
median (range)	198 (83.7–404.2)
**Baseline NLR**	
≤2.78	54 (40.9)
>2.78	78 (59.1)
**ΔNLR**	
≤0	82 (62.1)
>0	50 (37.9)
**Chemotherapy regimens**	
gemcitabine monotherapy	42 (31.8)
Gemcitabine and S1/capecitabine	15 (11.4)
Gemcitabine and nab-Paclitaxel	13 (9.8)
Gemcitabine and cisplatin/oxaliplatin	6 (4.5)
nab-Paclitaxel and S1	56 (42.5)

Abbreviations: KPS: Karnofsky Performance Status; WBC: White blood cell count; NLR: neutrophil to lymphocyte ratio; ΔNLR: ΔNLR was calculated by subtracting the baseline NLR from the NLR after 2 cycles of chemotherapy (cycle 2-cycle 0).

**Figure 2:**
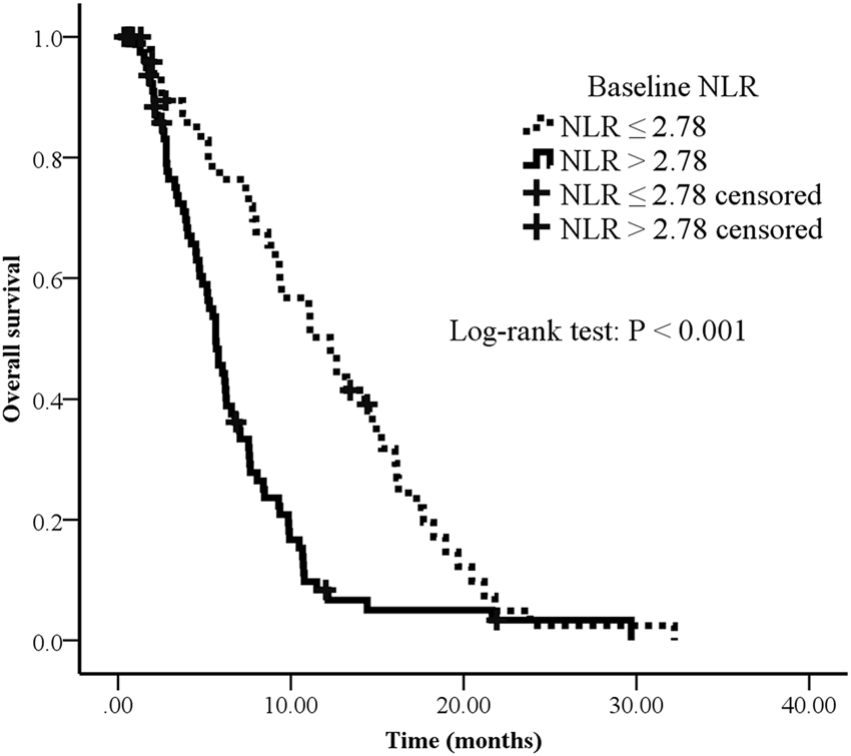
Overall survival curves in patients with advanced pancreatic cancer according to baseline NLR. Survival curves were estimated using Kaplan-Meier method. Total 132 patients, 116 passed away by July, 2016. The median OS of NLR > 2.78 and NLR ≤ 2.78 were 5.7 months (95% CI: 4.8–6.5) and 12.3 months (95% CI: 8.8–15.8), respectively.

**Figure 3:**
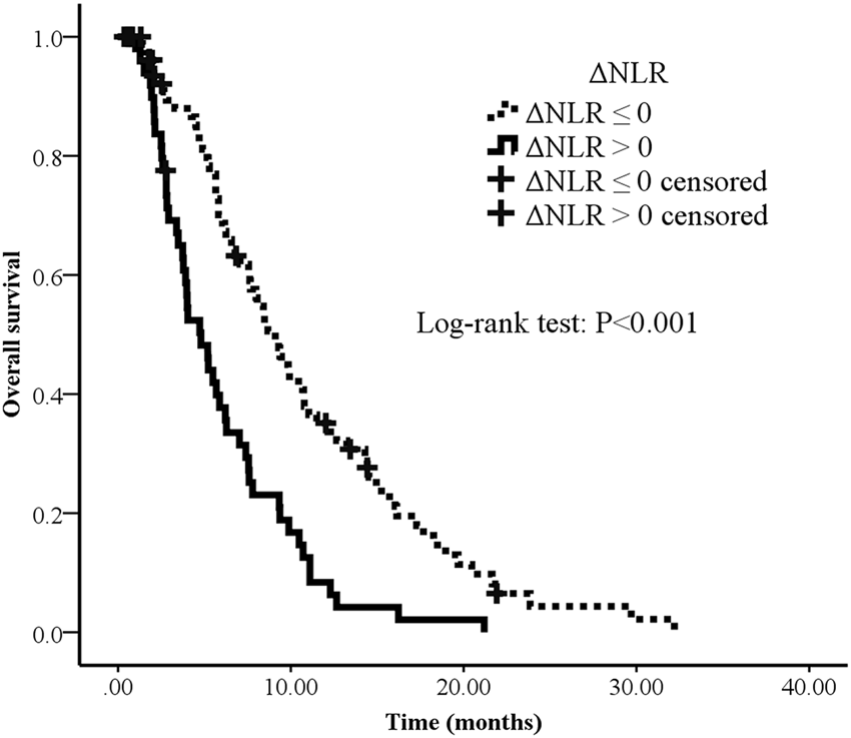
Overall survival curves in patients with advanced pancreatic cancer according to ΔNLR. Survival curves were estimated using Kaplan-Meier method. Total 132 patients, 116 passed away by July, 2016. The median OS of ΔNLR > 0 and ΔNLR ≤ 0 were 4.8 months (95% CI: 3.4–6.2) and 9.1 months (95% CI: 7.6–10.6), respectively.

Univariable and multivariable analysis was performed to investigate the prognostic role of baseline NLR and ΔNLR in pancreatic cancer patients underwent chemotherapy. Univariate analysis indicated male (P = 0.017), a high white blood cell (WBC) count after 2 cycles (P < 0.001), a high baseline NLR (P < 0.001) and ΔNLR > 0 (P < 0.001) were poor prognostic factors for overall survival (OS) in this study cohort (Figs 2, 3). Age, location, stage at diagnosis, albumin, KPS, and WBC, platelets and hemoglobin at baseline, platelets and hemoglobin after 2 cycles were not significantly associated with prognosis (Table 2).

**Table 2:** Univariate analysis for the association between clinical characteristics and survival in advanced pancreatic cancer patients.

Characteristics	N (%)	HR	95% CI	P value
**Gender**				
Female	47 (35.6)	1	reference	
Male	85 (64.4)	1.616	1.098–2.379	0.017
**Age**				
≤60	91 (68.9)	1	reference	
>60	41 (31.1)	0.900	0.607–1.335	0.601
**KPS**				
70	4 (3)	1	reference	
80	20 (15.2)	0.459	0.155–1.359	0.159
90	108 (81.8)	0.389	0.141–1.071	0.068
**Location**				
Head	46 (34.8)	1	reference	
Body/tail	86 (65.2)	0.977	0.846–1.129	0.755
**Stage at diagnosis**				
Locally advanced	31 (23.5)	1	reference	
Distant metastasis	101 (76.5)	1.468	0.952–2.265	0.082
**Albumin (g/l)**				
≤35	11 (8.3)	1	reference	
>35	121 (91.7)	1.287	0.400–1.507	0.455
**Baseline WBC**				
Increasing		1.049	0.991–1.110	0.102
**Baseline Hemoglobin**				
Increasing		1.001	0.991–1.011	0.838
**Baseline Platelets**				
Increasing		0.999	0.996–1.001	0.385
**WBC after 2 cycles**				
Increasing		1.078	1.045–1.111	<0.001
**Hemoglobin after 2 cycles**				
Increasing		0.999	0.991–1.007	0.876
**Platelets after 2 cycles**				
Increasing		0.999	0.997–1.001	0.427
**Chemotherapy**				
gemcitabine monotherapy	42 (31.8)	1	reference	
Gemcitabine and S1/capecitabine	15 (11.4)	0.698	0.385–1.265	0.236
Gemcitabine and nab-Paclitaxel	13 (9.8)	0.704	0.376–1.317	0.272
Gemcitabine and cisplatin/oxaliplatin	6 (4.5)	0.519	0.218–1.232	0.137
nab-Paclitaxel and S1	56 (42.5)	0.605	0.390–0.939	0.025
**Baseline NLR**				
≤2.78	54 (40.9)	1	reference	
>2.78	78 (59.1)	2.196	1.472–3.277	<0.001
**ΔNLR**				
≤0	82 (62.1)	1	reference	
>0	50 (37.9)	1.685	1.162–2.444	<0.001

Abbreviations: CI: confidence interval; HR: hazard ratio.

Multivariate analysis indicated that a high level of baseline NLR was a solid poor factor for OS (hazard ratio [HR] = 2.648, 95% CI: 1.631–4.300, P < 0.001) adjusted for gender, KPS, stage at diagnosis, and WBC, platelets, and hemoglobin at baseline and after 2 cycles. Whereas HR of death was 1.894 (1.160–3.091) for patients with ΔNLR > 0 compared to ΔNLR ≤ 0. Based on our analysis, baseline NLR and ΔNLR were independent prognostic factors for pancreatic cancer patients (Table 3).

**Table 3:** Multivariate analysis for the association between clinical characteristics and survival in advanced pancreatic cancer patients.

Characteristics	N (%)	HR	95% CI	P value
**Gender**				
Female	47 (35.6)	1	reference	
Male	85 (64.4)	1.334	0.876–2.033	0.158
**KPS**				
70	4 (3)	1	reference	
80	20 (15.2)	0.682	0.221–2.103	0.506
90	108 (81.8)	0.209	1.795–0.372	0.373
**Stage at diagnosis**				
Locally advanced	31 (23.5)	1	reference	
Distant metastasis	101 (76.5)	1.185	0.687–2.044	0.541
**Baseline WBC**				
Increasing		1.035	0.955–1.121	0.407
**Baseline Hemoglobin**				
Increasing		0.998	0.985–1.012	0.823
**Baseline Platelets**				
Increasing		1.000	0.996–1.004	0.923
**WBC after 2 cycles**				
Increasing		1.071	1.030–1.114	0.001
**Hemoglobin after 2 cycles**				
Increasing		1.004	0.995–1.013	0.420
**Platelets after 2 cycles**				
Increasing		0.997	0.995–1.000	0.057
**Chemotherapy**				
gemcitabine monotherapy	42 (31.8)	1	reference	
Gemcitabine and S1/capecitabine	15 (11.4)	0.757	0.351–1.634	0.479
Gemcitabine and nab-Paclitaxel	13 (9.8)	0.542	0.263–1.118	0.098
Gemcitabine and cisplatin/oxaliplatin	6 (4.5)	0.775	0.279–2.155	0.625
nab-Paclitaxel and S1	56 (42.5)	0.712	0.410–1.235	0.227
**Baseline NLR**				
≤2.78	54 (40.9)	1	reference	
>2.78	78 (59.1)	2.648	1.631–4.300	<0.001
**ΔNLR**				
≤0	82 (62.1)	1	reference	
>0	50 (37.9)	1.894	1.160–3.091	0.007

Abbreviations: CI, confidence interval; HR, hazard ratio.

The multivariate Cox regression model adjusted for gender, KPS, stage at diagnosis, and WBC, platelets, hemoglobin, at baseline and after 2 cycles, baseline NLR and ΔNLR.

**Figure 4:**
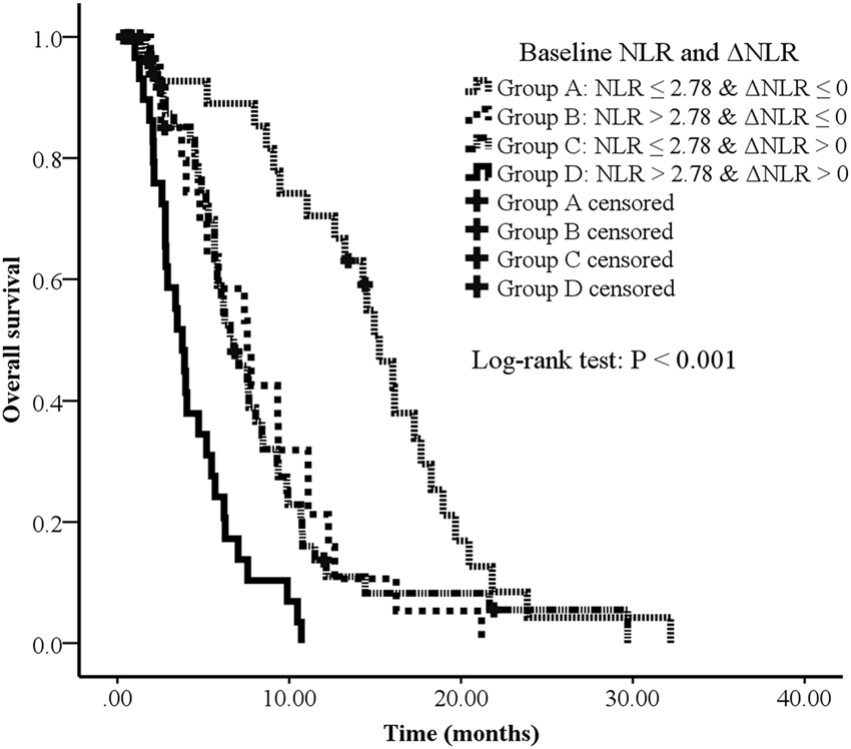
Overall survival curves in patients with advanced pancreatic cancer according to 4 risk groups (combining baseline NLR and ΔNLR). Survival curves were estimated using Kaplan-Meier method. Total 132 patients, 116 passed away by July, 2016. The median OS of group A, B, C and D were 15.2 months (95% CI: 13.2–17.2), 7.6 months (95% CI: 4.9–10.2), 6.8 months (95% CI: 5.1–8.4) and 3.8 months (95% CI: 2.9–4.7), respectively.

Combining both baseline NLR and ΔNLR, we further categorized patients into 4 groups: group A (NLR ≤ 2.78 & ΔNLR ≤ 0), group B (NLR > 2.78 & ΔNLR ≤ 0), group C (NLR ≤ 2.78 & ΔNLR > 0) and group D (NLR > 2.78 & ΔNLR > 0). Patients in group B (HR = 2.189, 95% CI: 1.097–4.371, P = 0.026), group C (HR = 2.733, 95% CI: 1.510–4.947, P = 0.001), and group D (HR = 5.817, 95% CI: 2.862–11.821, P < 0.001) had poorer prognosis (OS) compared to group A by multivariate analysis adjusted for gender, KPS, stage at diagnosis, and WBC, platelets, and hemoglobin at baseline and after 2 cycles (Table 4) (Fig. 4).

**Table 4:** Multivariate analysis for the association between 4 groups (combining baseline NLR and ΔNLR) and survival in advanced pancreatic cancer patients.

Group		N	OS (month)	HR	95% CI	P value
A	NLR ≤ 2.78 & ΔNLR ≤ 0	33	15.2	1	reference	
B	NLR > 2.78 & ΔNLR ≤ 0	21	7.6	2.189	1.097–4.371	0.026
C	NLR ≤ 2.78 & ΔNLR > 0	49	6.8	2.733	1.510–4.947	0.001
D	NLR > 2.78 & ΔNLR > 0	29	3.8	5.817	2.862–11.821	<0.001

Abbreviations: CI, confidence interval; HR, hazard ratio.

The multivariate Cox regression model adjusted for: gender, KPS, stage at diagnosis, and WBC, platelets, hemoglobin, at baseline and after 2 cycles.

## Discussion

On the basis of our data, we found the independent prognostic value of baseline NLR and ΔNLR in advanced pancreatic cancer. Several studies indicated the poor survival was associated with high baseline NLR of the cancers in lung^9, 10^, colon^11^, gastric^7^, breast^12^, ovarian^13^, prostate^14^ and pancreas^15–17^. Our findings of baseline NLR was in consistent with previous studies. In addition, we found that HR of death were significantly higher in patients with ΔNLR > 0 compared to ΔNLR ≤ 0 (HR = 1.807, 95% CI: 1.202–2.714, P = 0.007). Baseline NLR and ΔNLR in patients underwent chemotherapy appear to be of significant clinical value. Combining both baseline NLR (≤2.78 or >2.78) and ΔNLR (≤0 or >0), group D (baseline NLR >2.78 & ΔNLR >0) had a significant higher risk (HR = 5.817, P < 0.001) compared to group A (NLR ≤ 2.78 & ΔNLR ≤ 0), which is indicative of patients’ potential resistance to treatment and predicts poor survival.

NLR and other similar indicators, such as C-reactive protein, albumin, the Glasgow prognostic score, thrombocytosis and PLR may reflect systemic inflammation^18–20^. Until now, several theories regarding these facts have been made. A high NLR goes along with either a larger neutrophil amount or a relatively lower number of lymphocytes.

First, literatures showed neutrophils can irritate angiogenesis and suppress the immune system for anti-tumour activities to induce tumour growth^2, 21^. Several studies indicated that neutrophils were involved in the release of various cytokines and chemokines including vascular endothelial growth factor (VEGF) and matrix metalloproteinase (MMP)^22, 23^, which were important for tumour angiogenesis and metastasis^24, 25^. Fridlender *et al*.^26^ found that TGF-β within the tumour microenvironment induced a population of tumour-associated neutrophils (TAN) with a protumour phenotype. Lei Gong *et al*.^27^ confirmed that neutrophils played a very important role in the promotion of lung cancer which was strongly mediated through the IL-8/CXCR2 pathway and release of neutrophil elastase and development of a type 2 protumour microenvironment. *KRAS* mutation was the most common genetic alterations in pancreatic cancer^28^. Ji *et al*.^29^ indicated that activation of KRAS/RAF/MEK pathways could stimulate the accumulation of neutrophils, followed by intensive inflammatory response. Based on the above literatures, we think neutrophils could induce tumour growth and metastasis, and the increased number of tumour-associated neutrophils has been linked to poorer outcome in cancer patients. Similar findings in the literatures indicated that elevated neutrophils were significantly associated with larger tumour size and worse survival in patients with localized renal cell carcinoma^30^ and nasopharyngeal cancer^31^.

Second, *in vitro* studies showed that the cytolytic activity of lymphocytes and natural killer cells was suppressed when co-cultured with neutrophils, and the extent of suppression was proportionally enhanced to the addition of neutrophils^32^. Pillay *et al*.^33^ identified that neutrophil subpopulations could suppress T-cell proliferation by integrin Mac-1 and hydrogen peroxide. Lymphocytes were known to have a crucial role in tumour defense. Lymphocytes could induce cytotoxic cell death and inhibit tumour cell proliferation and migration^21, 34^. Therefore, a reduced lymphocyte count indicated a weaker immune reaction against tumour cells. Fogar *et al*.^35^ confirmed that lower total lymphocyte count in blood for patients with pancreatic cancer was associated with poor outcome. Therefore, the levels of baseline NLR and ΔNLR could reflect the inflammatory response and immune status of the patients undergoing chemotherapy, and theoretically both factors could be predictors for patients’ prognosis.

The limitations of this study included: (a) this was a single center retrospective study; (b) there were different chemotherapy regimens; (c) we had included limited sample size; and (d) the study was conducted only in the Chinese population.

In conclusion, we found both baseline NLR and ΔNLR were independent prognostic factors for patients with advanced pancreatic cancer underwent chemotherapy. Our results suggest that evaluation of baseline NLR and ΔNLR is helpful in prognosis prediction, drug dose adjustment and inflammatory status assessment. Further validation in a prospective study is warranted.

## Methods

### Patients

This was a retrospective study approved by the ethics committee of Chinese People’s Liberation Army (PLA) General Hospital. From January 1, 2010 to June 1, 2015, patients with advanced pancreatic cancer admitted for chemotherapy were included for analysis. Prior to chemotherapy initiation, written informed consent was reviewed and signed by the patients or their legal guardian. All relevant blood tests and treatments were performed based on institutional guidelines and regulations. Clinical data were retrieved and collected for retrospective analysis from the medical records of PLA General Hospital database electronically.

The inclusion criteria were: (1) patients were cytological or histologically confirmed pancreatic cancer and not eligible for operation; (2) patients received at least 2 cycles of chemotherapy (Please see Supplementary Table S1 for detailed information about chemotherapy regimens); (3) sufficient bone marrow function; (4) normal hepatic and renal function; (5) without targeted therapy or other biologics; (6) patients with a KPS score of 70 or more (on a scale from 0 to 100, with higher scores indicating better performance status); (7) no history of previous chemotherapy for advanced disease or adjuvant therapy within one year; (8) no radiotherapy. Exclusion criteria: (1) incomplete data of toxicities; (2) lost follow-up. Total 132 patients were eligible for analysis. Follow-up evaluations were performed every 3 months. Dates of death were obtained from the China disease prevention and control information system or telephone calls follow-up. Medical records were reviewed, and the cause of death was decided by investigator. Lost follow-up refers to the patient who was out of contact. We followed up until July 30, 2016 to obtain clinical and outcome information.

### Data collection

All relevant clinic-pathological data were retrieved from patient medical records. Laboratory data, including neutrophil and lymphocyte, WBC, platelets, hemoglobin were obtained within 1 week before chemotherapy and after 2 cycles of chemotherapy. The absolute neutrophil count was calculated by the percentage of segmented neutrophils out of the WBC count. The NLR was determined by the absolute neutrophil count divided by the absolute lymphocyte count. ΔNLR was calculated by subtracting the baseline NLR from the NLR after 2 cycles of chemotherapy (cycle 2-cycle 0). Overall survival was defined as the time from date of treatment to death. The primary study endpoint was OS. Censoring occurred if patients were still alive at last follow-up.

### Statistical analysis

Data were presented as median (interquartile) for continuous variables, and as frequency or percentage for categorical variables. Survival curves were estimated using the Kaplan–Meier method and compared by the log-rank test. Multivariate survival analyses were performed using Cox proportional hazards regression models. We used the ROC curve to determine the best cut-off values for OS with baseline NLR. All of the analyses were performed with the statistical software packages R (http://www.R-project.org, The R Foundation). Statistical significance was defined as a two-sided P < 0.05.

## Electronic supplementary material

supplementary information
