# Supplementary material for: Significance of baseline and change in neutrophil-to-lymphocyte ratio in predicting prognosis: a retrospective analysis in advanced pancreatic ductal adenocarcinoma
Source: Sci Rep. 2017 Apr 9;7:753. doi: 10.1038/s41598-017-00859-5 (PMC5429710; doi:10.1038/s41598-017-00859-5)
Supplement: Supplementary file 1 — supplementary information [file 41598_2017_859_MOESM1_ESM.pdf]

Significance of baseline and change in neutrophil-to-lymphocyte ratio in predicting prognosis: a retrospective analysis in advanced pancreatic ductal adenocarcinoma

Yang Chen, Huan Yan, YanRong Wang, Yan Shi*, GuangHai Dai*
Medical Oncology department 2, Chinese PLA General Hospital and Chinese PLA Medical School, Beijing, 100853, China
*Correspondence and requests for materials should be addressed to Y.S. (shibaiwan12@vip.sina.com) and G.H.D. (email: dgh19661007@126.com), YS and GHD contributed equally.

Table S1 Dosages of different chemotherapy regimens

Chemotherapy regimen	Dosage
gemcitabine monotherapy	Gemcitabine: 1000 mg / m^2, day 1, 8, every 3 – week;
Gemcitabine and S1 / capecitabine	Gemcitabine:1000 mg / m^2, day 1, 8<br>Three doses of S-1 were established according to the body surface area (BSA) as follows: BSA ≤ 1.25 m^2, 80 mg / day; 1.25 m^2 < BSA ≤ 1.5 m^2, 100 mg / day; and BSA > 1.5 m^2, 120 mg / day, day 1 - 14,<br>or<br>capecitabine: 1000 mg / m^2 twice daily on day 1 - 14, every 3 – week;
Gemcitabine and nab-Paclitaxel	Gemcitabine: 1000 mg / m^2, day 1, 8<br>nab-paclitaxel: 125 mg / m^2, day 1, 8, every 3 – week;
Gemcitabine and cisplatin / oxaliplatin	Gemcitabine: 1000 mg / m^2, day 1, 8<br>cisplatin: 60 mg / m^2 , day 1,<br>or<br>oxaliplatin: 130 mg / m^2, day 1, every 3 – week;
nab-Paclitaxel and S1	Nab-paclitaxel: 120 mg / m^2, day 1, 8;<br>Three doses of S-1 were established according to the body surface area (BSA) as follows: BSA ≤1.25 m^2, 80 mg /day; 1.25 m^2<BSA≤1.5 m^2, 100 mg /day; and BSA >1.5 m^2, 120 mg /day, day 1-14, every 3-week

KPS stands for Karnofsky Performance Status. A standard way of measuring the ability of cancer patients to perform ordinary tasks. The KPS scores range from 0 to 100. The higher the score meaning the better the patient is able to carry out daily activities.

100: Normal no complaints; no evidence of disease.

90: Able to carry on normal activity; minor signs or symptoms of disease.

80: Normal activity with effort; some signs or symptoms of disease.

70: Cares for self; unable to carry on normal activity or to do active work.

60: Requires occasional assistance, but is able to care for most of his personal needs.

50: Requires considerable assistance and frequent medical care.

40: Disabled; requires special care and assistance.

30: Severely disabled; hospital admission is indicated although death not imminent.

20: Very sick; hospital admission necessary; active supportive treatment necessary.

10: Moribund; fatal processes progressing rapidly.

0: Dead
